# It's a small world for parasites: evidence supporting the North American invasion of European *Echinococcus multilocularis*

**DOI:** 10.1098/rspb.2023.0128

**Published:** 2023-03-08

**Authors:** Maria A. Santa, Gerald Umhang, Claudia Klein, Danielle M. Grant, Kathreen E. Ruckstuhl, Marco Musiani, John S. Gilleard, Alessandro Massolo

**Affiliations:** ^1^ Department of Biological Sciences, Faculty of Science, University of Calgary, Calgary, Alberta, Canada; ^2^ Nancy Laboratory for Rabies and Wildlife, National Reference Laboratory for *Echinococcus* spp., Wildlife Surveillance and Eco-epidemiology Unit, ANSES, Technopôle Agricole et Vétérinaire, Malzéville, France; ^3^ Department of Clinical and Veterinary Clinical Sciences, Faculty of Veterinary Medicine, University of Calgary, Calgary, Alberta, Canada; ^4^ Friedrich-Loeffler-Institut, Bundesforschungsinstitut für Tiergesundheit, Federal Research Institute for Animal Health, Neustadt, Germany; ^5^ NORCE Climate, NORCE Norwegian Research Centre, Bjerknes Centre for Climate Research, Bergen, Norway; ^6^ Dipartimento di Scienze Biologiche, Geologiche e Ambientali (BiGeA), University of Bologna, Italy; ^7^ Department of Comparative Biology & Experimental Medicine, Host-Parasite Interactions (HPI) program, Faculty of Veterinary Medicine, University of Calgary, Calgary, Alberta, Canada; ^8^ Ethology Unit, Department of Biology, University of Pisa, Pisa, Italy; ^9^ UMR CNRS 6249 Chrono-environnement, Université Bourgogne Franche-Comté, Besançon, France; ^10^ Faculty of Veterinary Medicine, University of Calgary, Calgary, Alberta, Canada

**Keywords:** parasite invasion, *Echinococcus multilocularis*, North America, genetic characterization, allochthonous strains

## Abstract

*Echinococcus multilocularis* (*Em*), the causative agent of human alveolar echinococcosis (AE), is present in the Holarctic region, and several genetic variants deem to have differential infectivity and pathogenicity. An unprecedented outbreak of human AE cases in Western Canada infected with a European-like strain circulating in wild hosts warranted assessment of whether this strain was derived from a recent invasion or was endemic but undetected. Using nuclear and mitochondrial markers, we investigated the genetic diversity of *Em* in wild coyotes and red foxes from Western Canada, compared the genetic variants identified to global isolates and assessed their spatial distribution to infer possible invasion dynamics. Genetic variants from Western Canada were closely related to the original European clade, with lesser genetic diversity than that expected for a long-established strain and spatial genetic discontinuities within the study area, supporting the hypothesis of a relatively recent invasion with various founder events.

## 1. Introduction

In the current Anthropocene epoch, biological invasions, due to trans- and inter-continental movements, are generating a global biotic homogenization, influencing at the same time global patterns of disease [1,2]. Such invasions can lead to the introduction of novel hosts and their parasites, opening up opportunities for the emergence of diseases that can lead to outbreaks or the establishment of new endemism [3]. Indeed, many aetiological agents of emerging infectious diseases can be considered biological invaders [4]. As an example, the cestode *Echinococcus multilocularis* (*Em*), the causative agent of alveolar echinococcosis (AE), is considered an emerging or re-emerging disease in some regions, becoming an important public health concern in many countries worldwide [5,6]. This parasite is distributed across the northern hemisphere and is transmitted in sylvatic and semi-synanthropic cycles, involving small mammals as intermediate hosts (primarily arvicoline rodents) and canids as definitive hosts (e.g. red foxes (*Vulpes vulpes*), coyotes (*Canis latrans*) and dogs). In small mammals, the parasite invades and develops in internal organs (primarily the liver), being highly pathogenic. Therefore, the host either dies from the infection or is easily preyed on by the definitive hosts [6]. Conversely, wild canids only develop an intestinal infection, causing no substantial pathology [7]. Humans can act as dead-end hosts and develop AE by accidental ingestion of eggs via contaminated food, water or through contact with faeces of infected definitive hosts [8].

Although the geographical distribution of *E. multilocularis* is limited to the Northern hemisphere, its distribution and prevalence in wild and domestic hosts are increasing across its range, likely due to human activities [9–11]. The parasite has been reported in new areas previously considered non-endemic [12–15]. Furthermore, synanthropic hosts and domestic animals now have important roles in its transmission, contributing to the establishment of urban cycles [16,17]. Additionally, increased translocation of domestic and wild animals has generated new host species assemblages and introduced *E. multilocularis* (or particular genetic variants) into new areas [9,18,19].

Using concatenated sequences of three mitochondrial genes (*cox1*, *nad2* and *cob*), *Em* genetic variants have been grouped into four clades, mostly linked with their geographical origins: Asian, European, North American and one restricted to Mongolia [20]. However, Asian haplotypes have also been reported in Western Russia [21], Saint Lawrence Island (Alaska) [20] and, more recently, in central-eastern Europe (Poland) [22,23]. In addition, haplotypes clustering most closely with the North American clade have also been detected in Siberia [21]. In North America, the situation is similar, with genetic variants linked to geographically distant regions. In recent years, European-like haplotypes have been detected in Western Canada (Alberta, British Columbia, and Saskatchewan) in wild and domestic hosts [12,18,24–26]. Additionally, despite a historically low incidence of human AE in continental North America, with only two locally acquired cases ever reported (in 1923 and 1977, respectively), at least 17 cases have been described in the province of Alberta since 2013 [25,27], along with a case recently confirmed in the adjacent province of Saskatchewan [28], and more recently, two cases in the US [29,30]. Molecular characterization of six of the Albertan human cases implicated a European-like haplotype (ECA) that was also detected in local wild hosts [25,26]. Similarly, European-like haplotypes (different from those found in Canada) were identified in the two human cases in the US [29,30]. However, the distribution of European-like strains, their origin in North America, and potential interactions (e.g. competition or hybridization) with native North American genetic variants have not been elucidated. It has been suggested that the European strain could be more pathogenic and/or virulent than the North American strain, based on the historically low incidence of human cases of AE reported for the region. Consequently, it is of great importance to genetically characterize *E. multilocularis* in Western Canada, to assess the extent of the spread of European strains, their distribution in the main definitive hosts (coyotes and red foxes) and to elucidate potential sources of invasion of these strains.

In Canada, genetic characterization of *E. multilocularis* has been conducted primarily using mitochondrial (mtDNA) markers, yet these markers have relatively low variability. Conversely, the microsatellite EmsB, a multi-locus nuclear DNA marker, has greater discriminatory power than classic mtDNA markers, and has been used to identify spatio-temporal characteristics of *E. multilocularis* transmission in several European countries, at continental, national and local geographical scales [31–34]. Additionally, the combination of mitochondrial and nuclear markers has greater discriminatory power to identify genetic profiles and detect potential introgression events among genetic variants [22].

With this study, we aimed to: (i) assess the genetic diversity of *E. multilocularis* in Western Canada, based on EmsB profiles and mtDNA haplotypes; (ii) investigate genetic relationships with *E. multilocularis* isolates from other geographical regions across the globe, to identify possible sources of invasion; and (iii) evaluate the spatial distribution of the detected genetic variants to elucidate the spread dynamics of the European-like strains, possible spatio-temporal scenarios of its invasion process, and the likely origins of these European strains in Western Canada.

## 2. Materials and methods

### (a) Parasite collection and DNA extraction

*Echinococcus multilocularis* specimens were collected from gastrointestinal (GI) tracts of red foxes and coyotes of either road-killed or trap-harvested animals (trapped for purposes independent of this study), collected between 2012 and 2017 in Western Canada. Trapped animals were obtained from licensed trappers with the collaboration of the Alberta Trappers Association. GI tracts were screened using a modification of the scraping, filtration and counting technique, to identify and collect *Echinococcus* spp. worms [35,36]. We analysed *Em* worms from 70 coyotes and 13 foxes from northern, central and southern Alberta (AB); four coyotes from north-west British Columbia (BC); and 10 coyotes from southeast Saskatchewan (SK). Extraction of DNA was performed on up to five individual worms per host using the Nucleospin 96 Tissue Kit (Macherey-Nagel, Germany) for samples processed in France (Anses Nancy Laboratory for Rabies and Wildlife) and the E.Z.N.A. MicroElute Genomic DNA Kit (Omega Bio-tek, US) for samples processed in Canada (University of Calgary, Faculty of Veterinary Medicine). Extraction was performed following the manufacturer's instructions, and DNA was stored at −20°C until processed.

### (b) Genetic characterization using mtDNA

Genetic diversity of *E. multilocularis* was characterized by sequencing the genes *nad2* (1068 bp), *cob* (882 bp) and *cox1* (1608 bp). We used one to five worms per host, depending on worm availability and quality (intactness), using primers and PCR parameters as previously described [20]. Sequences obtained from the same *Em* worms from our previous studies were also included [25,26]. The sequences obtained per genetic locus were concatenated and aligned in Geneious 10.0.9 (Biomatters Ltd, New Zealand) and compared to nucleotide databases using the NCBI Nucleotide BLAST tool to identify the strain/haplotype (https://blast.ncbi.nlm.nih.gov). To analyse genetic relationships and possible origins of haplotypes identified in Western Canada, we constructed a haplotype network, based on Hamming distance, including reported European, Asian, Mongolian and North American haplotypes (based on full sequences of *cob*, *nad2* and *cox1*) [20,24,25,29]. R statistical software (R Development Core Team, 2022) and the package *pegas 0.14* [37] were used to construct the haplotype network. A phylogenetic tree was built via partitioned Bayesian analysis using MrBayes 3.2.7 [38], considering three partitions, corresponding to *cob*, *nad2* and *cox1*, and using *Echinococcus granulosus sensu stricto* (G1) as the outgroup. Reversible-jump Markov chain Monte Carlo analyses were run for 1 million generations, producing 10 000 trees, with the first 2,500 regarded as burn-in. The tree was plotted using the R-package *phangorn 2.3.1* [39].

### (c) Characterization of EmsB profiles

A fluorescent PCR assay was used to amplify the EmsB microsatellite, following a previous protocol [34]. Fragment analysis of PCR products was performed by capillary electrophoresis on an automatic sequencer (ABI Prism 310 and ABI 3500/3730; Life Technologies, CA). The resulting EmsB electropherograms were composed of several peaks between 209 and 241 bp. The size (base-pair length) and height (fluorescent signal intensity) of peaks present in each EmsB electropherogram were determined using GeneMapper 5.0 (Life Technologies, CA). Characterization of each EmsB profile was performed as described in the EmsB guidelines from the EWET database (https://ewet-db.univ-fcomte.fr/) [40]. Briefly, peaks below 10% of the sample's maximum peak height were considered artefacts and discarded. To normalize raw data, the height of each peak was divided by the sum of the height of all peaks of a given sample.

#### (i) Emsb genotyping, clustering and ordination analysis

Genotyping and hierarchical cluster analysis were performed by calculating the Euclidean distance between profiles and using the average link clustering method UPGMA [41]. The uncertainty of clusters was tested using multi-scale bootstrap resampling (1000 bootstrap replicates), obtaining approximately unbiased *p-*values (au) [42]. A dendrogram was built using *E. granulosus sensu stricto* (G1) as an outgroup. A distance threshold of 0.08 (average genetic distance observed after three generations in the rodent *Meriones unguiculatus*) was used to identify unique EmsB profiles; any branching below this threshold was considered genetically identical [43]. Unique EmsB profiles represented by only one sample were excluded from the analysis as they could not be technically validated [44]. To understand the structure of the clusters detected and unravel relational patterns among the genetic profiles, we performed a non-metric multi-dimensional scaling (NMDS) analysis based on the Euclidean distance matrix, using two and three dimensions to visualize the genetic distance between samples and their arrangement in reduced dimensions [45].

To evaluate genetic relationships with genotyped *E. multilocularis* isolates from Europe (historical and peripheral endemic areas), Asia (Japan, China and Kyrgyzstan) and North America (Canada, Alaska), each unique EmsB profile identified was compared to a reference world collection of profiles from the EWET database (https://ewet-db.univ-fcomte.fr; updated until 2017) [40] and from profiles reported after 2017 [33,44]. Profiles of 1275 samples from 17 countries were first compared to profiles obtained from our study; thereafter, the five profiles with the lowest genetic distance to each of our profiles (P1-P16) were used to build a dendrogram. Hierarchical cluster analysis was performed using R package *pvclust* [46] and NMDS was done using Primer & Permanova + add-on, Version 6 (PRIMER-E Ltd.).

#### (ii) Diversity analysis of EmsB profiles from Alberta

We evaluated the diversity of EmsB profiles in the province of Alberta (AB), the most extensively sampled area in our study. We divided this area into six geographical subregions: North-West (AB–NW), North-East (AB–NE), Central-West (AB–CW), Central-East (AB–CE), South-West (AB–SW) and South-East (AB–SE).

We used an integrated approach based on the framework of Hill numbers to assess: (i) sample completeness, (ii) asymptotic diversity estimates to infer true diversities, (iii) non-asymptotic standardization via rarefaction and extrapolation and (iv) evenness of each subregion (except for AB–NW due to low sample size), following the methodology described by Chao *et al*. [47]. First, to evaluate whether sample effort was sufficient and to assess the extent of undetected diversity, sample completeness was estimated for each subregion based on orders 0 ≥ *q* ≤ 2, where *q* is a number that determines the sensitivity of the measures to ‘species’ (i.e. EmsB profiles) abundances. Second, asymptotic diversity estimates were calculated using a sample size-based rarefaction and extrapolation sampling method [48]. The sample was extrapolated to double the size of the observed sample. If the curve stabilized and levelled off, then the asymptotic estimates were used to infer true diversity. Here, Hill numbers for order *q* ≥ 0 included the three most widely used diversity measures (i.e. Chao1 richness estimator, exponential Shannon diversity and inverse Simpson diversity, as special cases of orders *q* = 0, 1 and 2, respectively). Third, if data did not contain sufficient information to accurately infer the true diversity, this was inferred for a standardized sample coverage [49]. Here, we calculated non-asymptotic coverage-based rarefaction and extrapolation estimates for diversity orders *q* = 0, 1 and 2. The standardized sample coverage (i.e. equal fraction of an assemblage's individuals; *C*_max_) was selected as the minimum among the coverage values for samples extrapolated to double the size of the reference sample. Lastly, the evenness (i.e. distribution of EmsB profiles abundances) was calculated for each subregion. A bootstrap method (*n* = 100) was applied to obtain the associated 95% confidence intervals for all estimates.

Analysis of *β*-diversity was performed based on the Bray–Curtis dissimilarity index and complete linkage agglomerative clustering for hierarchical agglomeration [41]. Diversity of EmsB profiles in Alberta was compared to reported diversity in historical European endemic areas (i.e. Switzerland, Germany, Czech Republic, Austria and France) [31,50]. Diversity estimates were recalculated for these areas based on the standardized sample coverage (*C*_max_). Analyses were performed using the R package *iNEXT- 4steps* [47] and *vegan* [51].

#### (iii) Analysis of the spatial distribution of EmsB profiles

Alberta is divided into 174 wildlife management units (WMUs). Samples were collected from 38 WMUs and the location of each host was assigned as a random point within a radius of 15 km (minimum radius of a WMU) from the centroid point of the WMU where it was collected. This methodology was used because the exact geo-reference (latitude, longitude) for all the hosts was not available. A Mantel test was used to assess the hypothesis of genetic isolation by geographical distance, comparing a matrix of the genetic distance between EmsB profiles, and a matrix of the geographical distances between samples based on Euclidean pairwise distances [52]. A Mantel correlogram was used to investigate the underlying structure of the relationship and to measure the correlation between each class of distances [41]. A distance-based redundancy analysis (dbRDA), based on Bray–Curtis dissimilarity matrix of EmsB profiles, was performed using the two spatial variables (latitude and longitude) to estimate the proportion of the genetic variation explained by spatial structures [53]. Statistical significance of the model and variables was evaluated with a permutation test. Analyses were done using the R package *vegan* [51] and Primer & Permanova+add-on, Version 6 (PRIMER-E Ltd.).

## 3. Results

### (a) Genetic relationships of mtDNA haplotypes from North America supported the hypothesis of multiple invasion events of European origin

**Figure 1.**
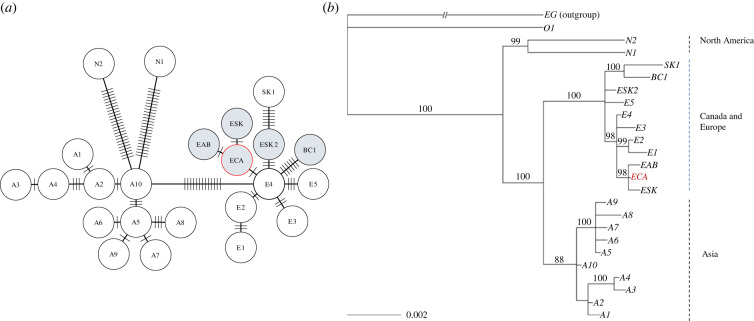
Genetic relationships among haplotypes of *Echinococcus multilocularis* from Western Canada and other historically endemic regions. (*a*) Mitochondrial haplotype network based on Hamming distance, including European, Asian and North American haplotypes previously reported (white circles) [20,24,25], compared to haplotypes from Western Canada detected in our study (grey circles). The network was constructed based on concatenated sequences of mitochondrial genes *nad2*, *cob* and *cox1*. The ECA haplotype (in red) is the most prevalent in Alberta and has been associated with the most recent cases of human AE in this province. (*b*) Phylogenetic tree inferred by partitioned Bayesian analysis performed on concatenated mitochondrial DNA, using *E. granulosus* (G1) as an outgroup. Values on tree nodes are Bayesian posterior probabilities. The 0.002 scale bar denotes genetic distance (nucleotide substitutions per site).

To characterize the genetic diversity of *E. multilocularis* based on mtDNA, we analysed 96 concatenated sequences of *cox1*, *nad2* and *cob* genes in adult worms recovered from 13 foxes and 77 coyotes. Virtually all (93/96) were identified as the previously described European-like haplotypes ECA, EAB, ESK, ESK2 and BC1 [25,26], and only three as the North American haplotype N2 (in two coyotes and one fox). Furthermore, the per cent identity to previously described haplotypes was 100%, with no new SNPs identified. The haplotypes ECA, EAB and N2 were present only in Alberta; ESK2 was in Saskatchewan and Alberta; ESK only in Saskatchewan; and BC1 only in British Columbia. The ECA haplotype was the most prevalent, with a frequency of 78.1% (75/96), followed by ESK2 with a frequency of 11.5% (11/96). Haplotypes BC1, EAB and N2 had a frequency of 3.1% (3/96) each, and only one sample was identified as ESK. The haplotype network showed that the European-like haplotypes found in Western Canada were closely related to the European clade, with few mutational steps (range, 1–6) between them, and that these were more similar to the European haplotype E4 (AB461395.1, AB461404.1, AB461414.1) than any other, suggesting a recent origin derived from the European clade. The most genetically distant haplotype was one from British Columbia (BC1), with six mutational steps to E4, followed by one from South-East Alberta and Saskatchewan (ESK2). Conversely, the haplotypes ECA, EAB and ESK were closely related, differing by only one or two mutations, having the lowest number of mutational steps to E4 (figure 1*a*), indicating a possible stem from a single invasion event. The phylogenetic tree confirmed the close relationship between the European-like haplotypes in Western Canada and the original European clade. Within this clade, the three similar haplotypes (EAB, ECA and ESK) were in the same branch with the E4, whereas BC1 was in another (along with the haplotype SK1), and more deeply branched, indicating a distinct most recent common ancestor. These branching nodes were supported by the Bayesian posterior probabilities (figure 1*b*).

### (b) Cluster and ordination analysis of EmsB profiles from Western Canada confirms the invasion hypothesis

**Figure 2.**
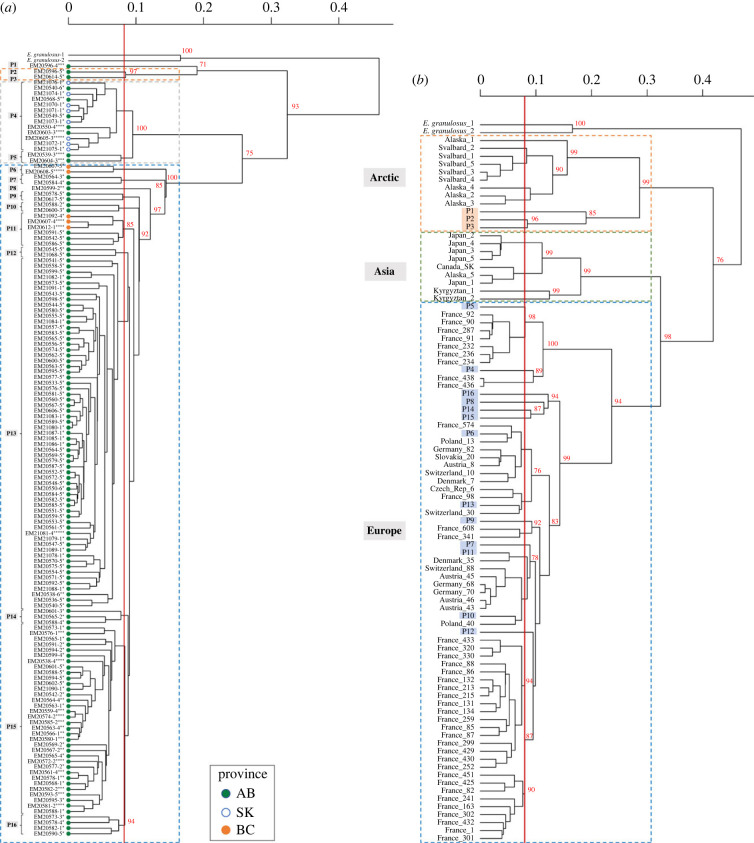
Cluster analysis of EmsB profiles of *Echinococcus multilocularis* from Western Canada and correspondence with the most similar profiles globally. (*a*) Dendrogram constructed by hierarchical clustering analysis based on EmsB genotypic data from *E. multilocularis* worms collected in Alberta (AB), Saskatchewan (SK) and British Columbia (BC) from 2012 to 2017. Two samples of *E. granulosus sensu stricto* (G1) were used as an outgroup. Approximately unbiased *p*-values (red numbers on nodes, in per cent) were calculated with multi-scale bootstrap resampling (1000 bootstrap replicates). A genetic distance threshold of 0.08 (red line) was used to identify unique profiles. Worms for the same host with indistinguishable profiles were pooled and the number of asterisks represents the number of worms for each profile. In total, 16 unique profiles were identified and grouped in three clusters based on unbiased *p-*values (sig. *α* = 0.05). (*b*) Dendrogram constructed with EmsB profiles from Europe, Asia and North America (from the EWET database), and the 16 EmsB profiles identified in our study in Alberta (orange and blue for North American and European clades, respectively). In total, 1275 samples from 17 countries were compared with the profiles obtained from our study; thereafter, five profiles with the lowest genetic distance to each of our profiles (P1–P16) were used to build the dendrogram.

Through fragment analysis of the multi-locus EmsB microsatellite, 204 individual EmsB profiles were computed and technically validated [40] (doi:10.6084/m9.figshare.16818607). Comparing the similarity of these 204 profiles, we identified 16 unique EmsB profiles based on the 0.08 threshold average genetic distance. These profiles formed three significant clusters: the first with samples from AB only, the second with samples from AB and SK, and the third with samples from AB and BC (figure 2*a*).

Two profiles (P13 and P15; exclusively from AB) were the most frequent, each representing 32.8% of the worms. When comparing the two definitive hosts, profile P13 was present in 69% of foxes and 64% of coyotes, and was the most prevalent in both hosts, representing 56% and 45% of worms in foxes and coyotes, respectively. Profile P4, the third most common (11.8%), was present in AB and SK. Profile P11 was only in BC and AB, corresponding to 5.9% of the worms. The other profiles represented 1–3.4% of worms and were primarily from AB (electronic supplementary material, table S1).

When comparing our profiles with profiles from the EWET world database (1275 samples, from 17 countries) to evaluate genetic relationships with other *E. multilocularis* isolates, 13 of our 16 unique profiles (81.2%) clustered with isolates from Europe, with eight being genetically indistinguishable (based on the 0.08 threshold) from profiles present in several European countries (i.e. Germany, France, Poland, Austria, Switzerland, Denmark, Slovakia and Czech Republic), and only three profiles clustering with samples from the Arctic (i.e. Saint Lawrence Island (Alaska) and Svalbard archipelago (Norway); figure 2*b*; electronic supplementary material, table S2).

**Figure 3.**
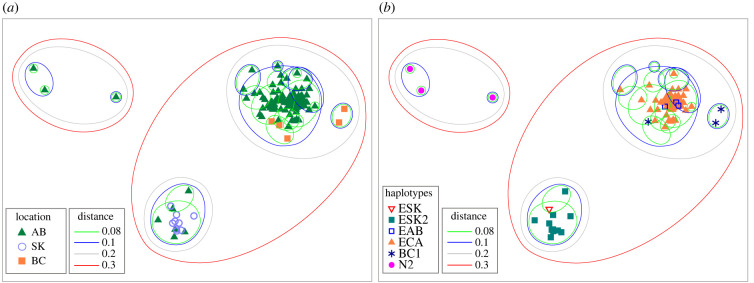
NMDS ordination plot of EmsB profiles of *Echinococcus multilocularis* collected in Western Canada from 2012 to 2017. Genetic distance was calculated based on the Euclidean distance. Coloured symbols represent the location (*a*) and mtDNA haplotype (*b*); two-dimensional stress = 0.05.

When the NMDS ordination analysis was performed, the representation of the genetic distances between profiles in two dimensions had the lowest stress (0.05; indicating the best representation), with the three clusters identified being linked with the location (i.e. provinces) (figure 3*a*) and the mtDNA haplotypes (figure 3*b*).

### (c) Genetic diversity of EmsB profiles in Alberta was lower than would be expected in endemic areas

The sample completeness profiles increased with diversity order (*q* ≥ 0), implying that there was undetected diversity (mostly less abundant EmsB profiles) in all subregions (except for AB–CW), and nearly all abundant EmsB profiles had been detected. The areas with the lowest sample coverage (*q* = 1) were AB–NE and AB–CE (89%) and the area with the highest coverage was AB–CW (100%). When Alberta was analysed as a single area, the sample completeness profile was 100% for all diversity orders (electronic supplementary material, table S3).

The size-based rarefaction and extrapolation analysis revealed that for each subarea the sampling curve stabilized for orders *q* = 1 and 2, but not for *q* = 0 (electronic supplementary material, figure S1), implying that the asymptotic diversity estimates could be used to infer true diversities (Shannon and Simpson), but not to estimate true genetic richness. When comparing the asymptotic estimates for *q* = 1 and 2 (electronic supplementary material, table S3), the highest diversity indices were found in AB–SW (5.9, 4.5) and the lowest in AB–CW (2.5, 2.4) and AB–SE (3.1, 2.2), with similar values for the other subregions.

**Figure 4.**
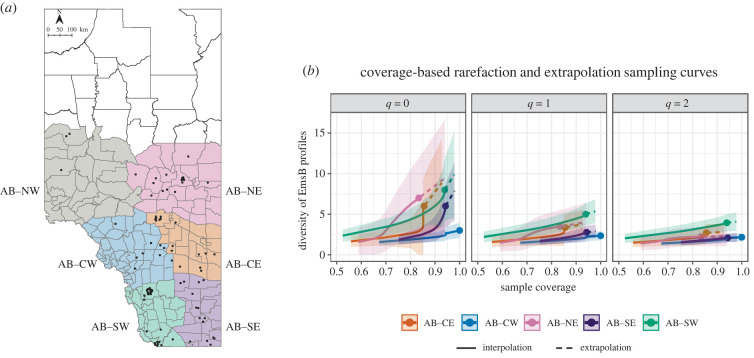
*Echinococcus multilocularis* samples collected in Alberta from 2012 to 2017, and coverage-based rarefaction and extrapolation sampling curves per subregion in Alberta, Canada. (*a*) Subregions in Alberta: North–West (AB–NW), North–East (AB–NE), Central–West (AB–CW), Central–West (AB–CE), South–West (AB–SW) and South–East (AB–SE). The dots represent the geographical locations of the parasite samples analysed. (*b*) Non-asymptotic coverage-based rarefaction (solid lines) and extrapolation (dashed lines) curves for orders *q* = 0, 1 and 2*,* (i.e. Chao1 richness, Shannon and inverse Simpson diversity) for each subregion in Alberta. Confidence intervals (95%) are represented by shading. Solid dots denote the observed data points. (AB–NW was excluded from the analysis due to the small sample size).

Since data were insufficient to infer the true richness, non-asymptotic coverage-based rarefaction and extrapolation curves were calculated (figure 4) and measures were computed up to a standardized coverage value of *C*_max_ = 94.5% (electronic supplementary material, table S3). For this *C*_max_, the corresponding highest richness estimate (*q* = 0) was found in AB–CE (8.2) and the lowest in AB–CW (2.7). For evenness profiles, all values of *q* showed the highest evenness of EmsB profiles in AB–CW and the lowest in AB–SE.

Analysis of *β*-diversity was performed by constructing a dendrogram based on the Bray–Curtis distance to compare genetic diversity between areas. There was a higher similarity between the central and northern regions, and a second branch grouping for the southern regions (electronic supplementary material, figure S2), with low dissimilarity among all regions (0.27 to 0.53). When comparing non-asymptotic diversity indices from subregions in Alberta with diversity reported in historical endemic areas from Europe (recalculated based on *C*_max_ = 94.5%), the range of values (min. and max.) for all *q* orders was higher in European countries than in most subregions in Alberta. Moreover, when Alberta was analysed as a single area, estimates for orders *q* = 1 and *q* = 2 were lower than in all areas in Europe (except for South Germany; electronic supplementary material, table S3). The same pattern was observed for the evenness profiles, with Alberta being the most uneven area (0.2–0.3), which indicates the presence of a dominant EmsB profile and low diversity.

### (d) Spatial distribution of EmsB profiles in Western Canada

**Figure 5.**
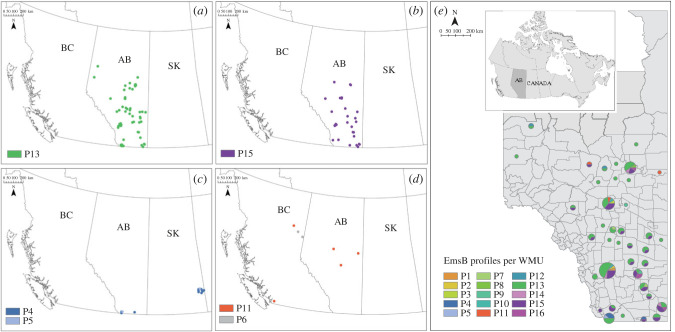
Spatial distribution of the EmsB profiles of *Echinococcus multilocularis* collected in Western Canada from 2012 to 2017. (*a*,*b*) The distributions of P13 and P15, the two main EmsB profiles in Alberta; (*c*) the distribution of profiles P4 and P5, present only in Alberta (AB) and Saskatchewan (SK); (*d*) the distribution of the profiles P6 and P11, present exclusively in BC and AB. (*e*) The distribution of EmsB profiles per WMU in Alberta. The sizes of the pie charts in (*e*) represent the numbers of samples collected.

Profiles were spatially clustered, with profiles P13 and P15 found exclusively in Alberta (figure 5), and those from BC (P6 and P11) mostly localized only there, except for P11, which was found also in northern AB, close to the BC border. Similarly, in southern SK, only P4 and P5 were detected, with both profiles also present in southern AB. No profiles from BC were detected in SK, or *vice versa*. To assess the hypothesis of genetic isolation by geographical distance, we used a Mantel test comparing a genetic distance matrix between EmsB profiles and a matrix of the geographical distances between samples, based on Euclidean pairwise distances [52]. There was no significant correlation between genetic and geographical distances when testing samples only from AB (*r* *=* 0.02, *p* = 0.30). However, the Mantel test yielded a significant correlation (*r* *=* 0.4, *p* < 0.001) when including samples from BC and SK in the analysis. The Mantel correlogram indicated a significant positive correlation (more similar samples) within 50 to 550 km, but a negative correlation (more dissimilar samples) when > 600 km apart (electronic supplementary material, figure S3*a*).

In a dbRDA using the two spatial variables, latitude and longitude, 21.1% of the variance of the genetic matrix was explained by geographical distance (for samples from all three provinces). Still, only the longitude—the axis dividing provinces—was significant (*F* = 30.36, *p* < 0.001) (electronic supplementary material, figure S3*b*).

## 4. Discussion

In this study, we investigated the origin of the European-like strains of *E. multilocularis* in Western Canada, their spread dynamics and potential spatio-temporal scenarios of their process of invasion in North America. We assessed the genetic diversity of the parasite in this region, comparing EmsB profiles and mtDNA sequences; most genetic variants from Western Canada were closely related to the original European clade. Furthermore, the diversity of the European genetic variants found in Western Canada was low compared to that expected for a long-established endemic strain, supporting the hypothesis of relatively recent introductions and discounting the hypothesis of North America as an ancestral endemic focus for this strain. Moreover, the spatial analysis indicated genetic discontinuity, only evident over large geographical scales, suggesting various introduction events in Western Canada.

When using mtDNA as a genetic marker, most haplotypes detected were closely related to the European clade, with few point mutations from European isolates [18,24–26]. However, the number of European samples with sequences available for the three mtDNA genes was limited. Therefore, although not likely, it cannot be ruled out that haplotypes from Western Canada may also be present in European countries but not yet detected. The Bayesian phylogenetic tree implied different evolutionary origins and pathways of the haplotypes found in Western Canada. The ECA, EAB and ESK were grouped in the same branch, whereas BC1 was in a different branch and showed a higher divergence with the closest haplotype SK1. This division was also evident in their geographical distribution, as BC1 was detected in British Columbia, but not in Saskatchewan, whereas ECA and EAB were only in Alberta. These results suggest different spatio-temporal scenarios, with multiple introductions of the European strains to Western Canada, possibly occurring at different times in the past centuries, causing worm populations to be more or less isolated. Indeed, various sources of invasion have been proposed, including the translocation of domestic dogs from European endemic areas [12], introduced European red foxes imported for sport hunting [54], and/or introduction of the parasite via translocation of intermediate hosts with international shipping [9].

During the process of invasion, the likelihood of successful establishment will strongly depend on the propagule pressure (i.e. the number of introduction events and the number of infective stages released). Therefore, the repeated release of a large number of individuals in multiple locations can facilitate the long-term establishment and increase the rate of spread of non-native populations [55]. However, multiple introductions and high genetic variation do not seem to be indispensable for a successful invasion [56]. For a parasite like *E. multilocularis*, reproductive traits (i.e. hermaphroditism, asexual multiplication in intermediate hosts, self- and cross-fertilization and development of thousands of sexually mature adult worms in each definitive host) could have also been determinant in the first stages of invasion, boosting local spread, but producing in turn geographical isolation of the different genotypes introduced.

The EmsB microsatellite enabled detection of more profiles (16 in total) in comparison to only seven mtDNA haplotypes, helping to identify local genetic variation and trace the invasion process of the parasite. When compared to a world database, most of the profiles we found (13 of 16) clustered within the European group, having a low genetic distance to European isolates (ranging from 0.05 to 0.1). Moreover, half of the profiles were genetically indistinguishable from known European ones, supporting the hypothesis of a recent introduction of these European genetic variants. By contrast, the remaining three EmsB profiles (P1–P3), which were genotyped as North American haplotype N2 based on mtDNA, clustered within the Arctic clade and were genetically more distant from the Alaskan and Svalbard isolates (0.21 to 0.42), suggesting a more prolonged genetic isolation, consistent with the greater divergence between the haplotypes N1 and N2 [20,57].

Complete agreement between the two markers, in correlation with the geographical location, is not guaranteed. Umhang *et al.* [22] described samples from Poland clustering within the European clade, based on mtDNA, whereas they were genotyped as Asian-type profiles using EmsB, suggesting some degree of introgression between the two strains. Regardless, these two markers facilitated differentiation between North American and European genetic variants, with no evidence of introgression between them. All EmsB profiles clustered within the European clade were identified as European-like haplotypes based on mtDNA, whereas EmsB profiles identified as North American strains were in a different cluster. Moreover, most mtDNA haplotypes were represented by more than one EmsB profile from the same cluster, with samples from Saskatchewan clustering apart from samples from British Columbia, as observed with mtDNA haplotypes.

In Europe, the expansion history of the parasite from the historical endemic core area into peripheral regions has been governed by a mainland–island system of transmission, in which the ancestral focus in central Europe served as a ‘mainland’ supplying the peripheral areas (islands), perhaps due to dispersals of fox populations. From a genetic perspective, this resulted in invasion events with introduction of only a few genetic profiles, resulting in low genetic diversity in the colonized region compared to greater diversity in the ancestral endemic foci [33,50,58]. In our study, the genetic diversity indices recalculated for Switzerland (endemic area), based on *C*_max_ = 94.5%, were as high as 15 (*q* = 1) and 11.5 (*q* = 2), with an estimated richness of 23 profiles. In comparison, in the province of Alberta (with 661 848 km^2^ and almost twice as big as Germany), only 15 EmsB profiles were found, with genetic diversity indices between 2.3 to 5.1 (*q* = 1) and 2.1 to 4.1 (*q* = 2) in the five subareas, indicating low genetic diversity. This is inconsistent with the hypothesis of an undetected historical endemic area of the European-like clade. Moreover, the genetic indices recalculated for Switzerland were more than three times higher than those calculated for all of Alberta.

In this Canadian province, the highest genetic richness was found in the central east region, and the highest genetic diversity based on the abundance of each EmsB profile was found in the southwestern region. However, the degree of differentiation between *E. multilocularis* populations from all five subregions was low, with widespread distribution of two profiles (P13 and P15) representing 77% of the samples from this province. Moreover, these two profiles might be considered as part of one population, due to: (i) the low genetic distance between them (0.14), (ii) their overlap in geographical distribution and (iii) the relatively high frequency of co-occurrence in the same host (19 hosts). These results were consistent with previous studies using mtDNA showing a higher prevalence and wider geographical distribution of the haplotype ECA, the causative agent of the most recent human cases of AE in Alberta [25,26]. Therefore, the presence of a single predominant genetic variant in Alberta supported the hypothesis of a single invasion event responsible for the initial establishment of a small number of *E. multilocularis* individuals in that province.

The subsequent spread across the province was likely aided by red foxes, but mostly by the most abundant host, the coyote, which has a larger home range (greater than 100 km^2^) and higher dispersal distances (up to 300 km) [59–62]. In a previous study comparing coyotes and red foxes from Alberta infected with the ECA haplotype, the difference in intensity of infection (worm burden) between both hosts was significantly higher in coyotes than foxes [26], which could be related to a lack of co-evolved resistance in coyotes to the European strains. Therefore, the coyote, being a naïve host, might be the primary source of environmental contamination with eggs of these strains. In this study, we did not perform an analysis of intra-host diversity to compare red foxes and coyotes due to the low sample size of red foxes. However, we observed a higher prevalence of the profile P13 (identified as the ECA haplotype based on mtDNA) in both coyotes and red foxes, and a low prevalence of North American profiles, which is consistent with previous studies using deep amplicon sequencing [26]. Further analyses of differences in the prevalence and intensity of infection of European and North American genetic variants in coyotes compared to red foxes will be important to understand the role of the two hosts in the transmission and spread of the European strains and the intra-host competition between North American and European strains.

To estimate the expansion rate of the parasite, Takumi *et al*. [63] used a mathematical model predicting a spreading rate of 2.7 km per year, from an endemic to a non-endemic area in the Netherlands. By contrast, the presence of highly vagile hosts, like coyotes in Western Canada, could have facilitated and accelerated the spread of the European strain. Thus, it is likely that the process of colonizing the entire province of Alberta was completed in a shorter time frame, expanding from the south to the north, as evidenced by the highest differentiation between the Southern and the Northern regions of Alberta, and the distribution of the least common profiles in distant regions. This pattern, with one single cluster of profiles being the most prevalent, and low genetic diversity, was similar to that found in European regions initially not considered endemic. For example, in a survey conducted in Poland, only one profile was the most prevalent and was distributed across the country [33]. Similarly, in Denmark and Sweden, one profile represented 68.4 and 55.5% of worms, respectively [44].

In a previous study using EmsB to assess the genetic diversity of the parasite at a continental scale in Europe [50], only 5% of the genetic variability was explained by geographical distance. In our study, there was no significant correlation when analysing samples from Alberta alone. However, there was a significant correlation when including samples from British Columbia and Saskatchewan, with more dissimilar samples that were far apart (greater than 600 km). In the distance-based redundancy analysis, 21.1% of the genetic variability was explained by geographical distance, with longitude being the only significant spatial variable. Our results could indicate independent invasion events in each province and isolation by distance, with the exchange of some genetic variants between neighbouring regions, thanks to the dispersal movement of definitive hosts. Furthermore, these invasion events were likely influenced by geographical barriers, e.g. the Rocky Mountains between Alberta and British Columbia.

Despite the smaller sample size in Saskatchewan and British Columbia, our results were consistent with previous studies using mtDNA characterization in these areas. In British Columbia, Gesy *et al*. [18] reported only one European-like haplotype (BC1) in the immediate area of Quesnel, BC. Likewise, in a study in Saskatchewan, only one European-like haplotype (SK1) was detected in the central area of the province. Yet, seven haplotypes that belonged to the North American clade were detected in the southern region [24]. However, in our study in Alberta, the abundance of the North American strain was surprisingly low, compared to the European strains. Nonetheless, the genetic variability of the North American strain (i.e. number of disparate profiles in a few hosts) was high (three EmsB profiles in eight worms from two hosts), which is consistent with what would be expected from an ancestral endemic strain. The difference in the prevalence, genetic diversity and distribution between the North American and the European strains observed in Alberta, using nuclear and mitochondrial markers, was relevant, suggesting some degree of competitive interactions, with a population expansion of the European over the North American strain [26].

This was the first large-scale study using both nuclear and mitochondrial markers to assess the genetic diversity of *E. multilocularis* in North America. The use of EmsB improved characterization of parasite diversity, even at a fine geographical scale, whereas the use of mtDNA aided in unravelling the evolutionary process of the invasion of the European strain. As evidenced in this and previous studies, the implementation of genetic population structure analysis is a powerful tool to trace the origins and history expansion of parasites and their hosts in the areas invaded and for the assessment of public health risks. For example, a genetic study of raccoons (*Procyon lotor*) in the Netherlands (where they were previously absent) and their GI nematode parasite (*Baylisascaris procyonis*) showed that most of the Dutch raccoons and their roundworms were introduced through ex-captive individuals, which ultimately aided in the development of control measures of these invasive populations [64]. Recently, a genomic analysis allowed the evaluation of the process of colonization of the Americas by *Schistosoma mansoni*, a blood fluke that infects humans and that was introduced into the Americas from Africa during the Trans-Atlantic slave trade [65]. In this study, no evidence of population bottlenecks was observed, suggesting that *S. mansoni* parasites were pre-adapted to the Americas and able to establish with relative ease.

Further work on the study of genetic diversity of *E. multilocularis* including wild and domestic hosts from British Columbia and Saskatchewan, as well as from eastern Canadian provinces where the parasite has been recently detected, and in central US states, will be pivotal to understanding the current distribution and expansion trend of the European strains in continental North America, including the role of the main definitive hosts (coyotes and foxes) in harbouring and spreading these strains.

## Data Availability

All data generated or analysed during this study are included in this article (and its supplementary information files). The dataset of EmsB profiles is available in the Figshare repository: (https://doi.org/10.6084/m9.figshare.16818607) [66]. The data are provided in the electronic supplementary material [67].
